# Comparison of Two Cap Thickness in Small Incision Lenticule Extraction: 100μm versus 160μm

**DOI:** 10.1371/journal.pone.0163259

**Published:** 2016-09-21

**Authors:** Miao He, Wei Wang, Hui Ding, Xingwu Zhong

**Affiliations:** 1 Zhongshan Ophthalmic Center, State Key Laboratory of Ophthalmology, Sun Yat-Sen University, Guangzhou, People’s Republic of China; 2 Hainan Eye Hospital, Zhongshan Ophthalmic Center, Sun Yat-sen University, Haikou, Hainan Province, China; National Eye Institute, UNITED STATES

## Abstract

**Purpose:**

To compare the changes of biomechanical properties, endothelial cell density (ECD), and posterior corneal elevation (PCE) after femtosecond small incision lenticule extractions (SMILEs) with 100μm versus 160μm cap thicknesses.

**Methods:**

A total of 12 rabbits were randomly assigned into two groups of 6 each. SMILE was performed at a depth of either 160μm (160-cap group) or 100μm (100-cap group). Corneal biomechanics, PCE, ECD were evaluated pre-operatively, 1week, 1 month, 2 months, 3 months, and 4 months post-operatively by Pentacam, Corvis ST, in vivo confocal microscopy (IVCM) respectively. The Young’s modulus was obtained by strip-extensometry test 4 months after surgery.

**Results:**

At each time point, the second applanation time (A2T) was similar between the groups with the exception of 4 months after surgery (22.66±0.16 ms in the 160-cap group versus 21.75±0.29 ms in the 100-cap group, *p* = 0.004). Neither deformation amplitude (DA) nor the first applanationtime (A1T) were significantly different between the two groups. The postoperative posterior surface did not shift forward, the changes of PCE and ECD were not significantly different between the two groups at any observation time. Young’s modulus was higher in the 160-cap group than that in the 100-cap group with no statistical significance (P>0.05). Regression analyses showed that the PCE changes and Young’s modulus were not affected by cap thickness (CT) or residual stromal bed thickness (RBT) (All P>0.05).

**Conclusions:**

The differences of corneal biomechanics, posterior surface elevation, or ECD changes were quite small when using 100μm or 160μm cap thicknesses in SMILE.

## Introduction

Excimer laser surgery and femtosecond laser assisted lenticule extraction surgery are the main corneal refractive surgery for correcting myopia and myopic astigmatism. The small incision lenticule extraction (SMILE) is an “all-in-one” surgery by producing a stromal lenticule and extracting it through a 2.0–4.0 mm cut on the edge of the lenticule. It is well documented that SMILE is superior to other flap-related excimer laser surgeries since it can preserve more sub-basal corneal nerves and reduce damage to the biomechanical strength of the cornea.[1–3] However, one limitation of SMILE is the re-treatment in case of residual refraction. Re-treatment after SMILE is usually done with surface refractive surgeries such as Photorefractive Keratectomy (PRK), laser assisted in situ keratomileusis (LASIK) or femto-second assisted LASIK (FS-LASIK), whether secondary SMILE can be used for re-treatment remains elusive.

Experimental studies have shown that the anterior stroma of cornea is stiffer than the posterior stroma and plays an important role in sustaining the stability of corneal biomechanics. If SMILE is performed in a deeper location (thicker cap) more anterior corneal stroma over the lenticule will be preserved and the possibility of Bowman’s membrane being preserved will increase. Since the anterior stoma and Bowman’s membrane are essential in protecting corneal biomechanical strength, SMILE with a thicker cap will result in greater corneal tensile strength.[4] Reinstein et al. arrived at a conclusion after calculation using a mathematical model that a thicker cap in SMILE would increase total stromal tension (TTS) while a thicker flap in LASIK would decrease TTS. [5]In Egyptian myopic patients, it was reported that SMILE with deeper lenticule led to greater corneal hysteresis (CH) and corneal resistance factor (CRF), indicating greater stiffness of cornea.[6] Another advantage of SMILE with a thicker cap is that it leaves more space within the cap increasing the possibility for re-treatment using SMILE after a primary SMILE procedure.

However, a thicker cap in SMILE may be associated with two problems. First, a thicker cap will cause a thinner residual stroma bed, which is a risk factor of posterior ectasis.[7] Second, if the lenticule is closer to the posterior surface, whether the endothelial layer will be negatively affected remains unclear. In this prospective study, we aimed to investigate the corneal biomechanical characteristics by comparing the changes in corneal biomechanics, endothelial cell density (ECD), Young’s Modulus, and posterior corneal elevation (PCE) after performing SMILE at depth of 100μm versus 160μm for myopic corrections.

## Methods

### Experiment design

A total of 12 adult New Zealand white rabbits (Tianqing Co., Ltd, Changsha, China) were randomly assigned into two groups of 6 each using a computer-generated randomization schedule. Firstly, 12 random numbers were generated by SPSS software (version 20.0, Chicago, IL) with the random seed of 2000000. Secondly, these random numbers were ranked from smallest to largest. Thirdly, six rabbit of greater ranks were allocated into the first group (100-cap group) and the rest into the second group (160-cap group). The right eyes were selected for surgery. Both groups underwent a myopic treatment correction of -4.00 diopters (D). Central corneal thickness (CCT) and the PCE was obtained by corneal topography with a Pentacam-HR Scheimpflug camera (Oculus Optikgeräte GmbH, Germany). Thickness of cornea facing the center of the pupil was regarded as the CCT. The in vivo corneal biomechanical metrics were evaluated by Corvis ST (Oculus Optikgeräte GmbH, Wetzlar, Germany). The ECD was measured using in vivo confocal microscopy (IVCM) (HRT 3; Heidelberg Engineering GmbH,Heidelberg, Germany). Measurements of Pentacam, Corvis ST and IVCM were taken twice and the average results were analysed and presented. The recovery of the corneal incision was assessed by slit-lamp biomicroscopy. All the above examinations were conducted pre-operatively, 1week, 1 month, 2 months, 3 months and 4 months post-operatively. All examinations were completed by experienced technicians who were blind to the design of the study. At 4 months after surgery, the animals were sacrificed with an intravenous injection of an air overdose into the ear margin after anesthesia, then ex-vivo stress-strain test was conducted. The animals were treated according to the guidelines of the Association for Research inVision and Ophthalmology (ARVO) Statement for the Use of Animals in Ophthalmic and Vision Research. This study was approved by the Animal Ethics Committee of Hainan eye hospital, Zhongshan ophthalmic center, Sun Yat-sen University.

### SMILE procedure

The animals were anesthetized with ketamine hydrochloride (Gutian Pharmaceuticals Co., Ltd., Fuzhou, China) 20 mg/kg intramuscularly and subsequent 0.4% oxybuprocaine hydrochloride (Santen Pharmaceutical Co., Ltd., Osaka, Japan) instilled on the cornea surface every 2 minutes for overall three times before surgery according to the drug directions. An eyelid speculum was used to keep the eye open. A small-sized curved interface cone was utilized to dock on the centre of the cornea. The surgery was conducted under femtosecond laser system (Carl Zeiss Meditec, Jena, Germany). All surgeries were performed by the same surgeon (XZ). The posterior surface of the lenticule was cut in a “spiral in” pattern, the anterior surface and the cornea cap were cut in a “spiral out” pattern. A 2.5mm arcuate incision at 140° was made at the end. The interior and posterior surfaces of the lenticule were released with a femto-lamellar dissector (Asico, Westmont, IL) and then extracted using Tan DSAEK forceps (Asico). Laser energy was 28nJ, and the cap thickness was set 100μm for 100-cap group and 160μm for 160-cap group. The cap diameter was 7.5mm and the optical zone diameter was 6.5mm. The spot distance and trackingspacing were set at 4.5μm for the cap and lenticule, and 2 μm for the side cut. After surgery, the animals received levofloxacin (0.5%, Santen Pharmaceutical Co., Ltd., Osaka, Japan) combined with tobramycin and dexamethasone (Alcon Laboratories, Inc., Fort Worth, TX) eye drops three times a day for one week.

### IVCM imaging

A sterile polymethylmethacrylate cap (Tomo-Cap; Heidelberg Engineering) filled with a layer of 0.2% Carbomer Eye Drops (Dr. Gerhard Man,Chem.-Pharm. Fabrik GmbH, Berlin, Germany) was mounted in front of the cornea module, then the module was manually advanced until the cap contacted the central surface of the cornea. A section scan was used to record each layer from epithelial to endothelial. A region of interest (ROI) of about 0.06 mm^2^ was outlined for the analysis of the ECD. According to the manufacturer’s recommendation, at least 60 endothelial cells with clear frames in the ROI were marked and then the ECD was automatically calculated by the computer software.

### Corvis ST and Pentacam examination

**Fig 1:**
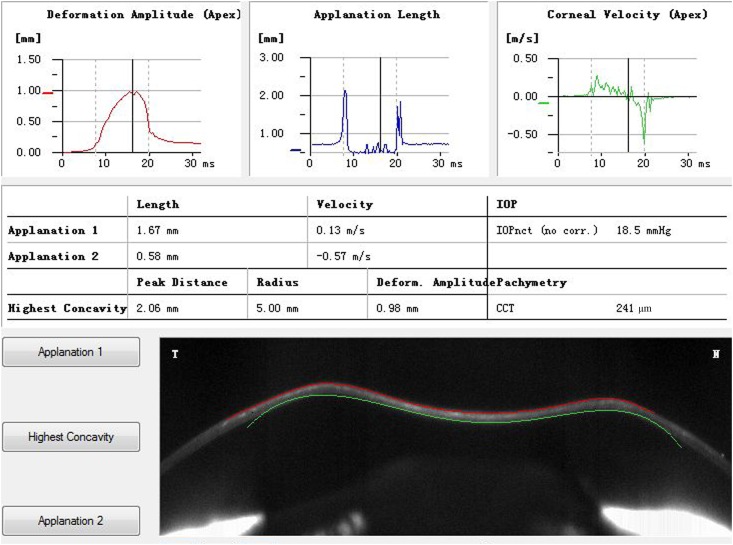
Corvis ST output parameters. IOP, intraocular pressure; CCT, central corneal thickness.

More than 10 corneal biomechanical parameters were provided by Corvis ST provides (Fig 1). Three parameters including deformation amplitude (DA), first applanation time (A1T), and second applanation time (A2T) were chosen for analysis in this study because they were demonstrated to be with highest reproducibility and repeatability. [8–13] A less deformable cornea was considered to reach A1 slower (with a longer A1T and A1L, and smaller A1V), to show smaller concavity (with smaller DA and higher PD and CCR), and to reach A2 faster (with a shorter A2T, longer A2L, and a higher A2V).[11]

**Fig 2:**
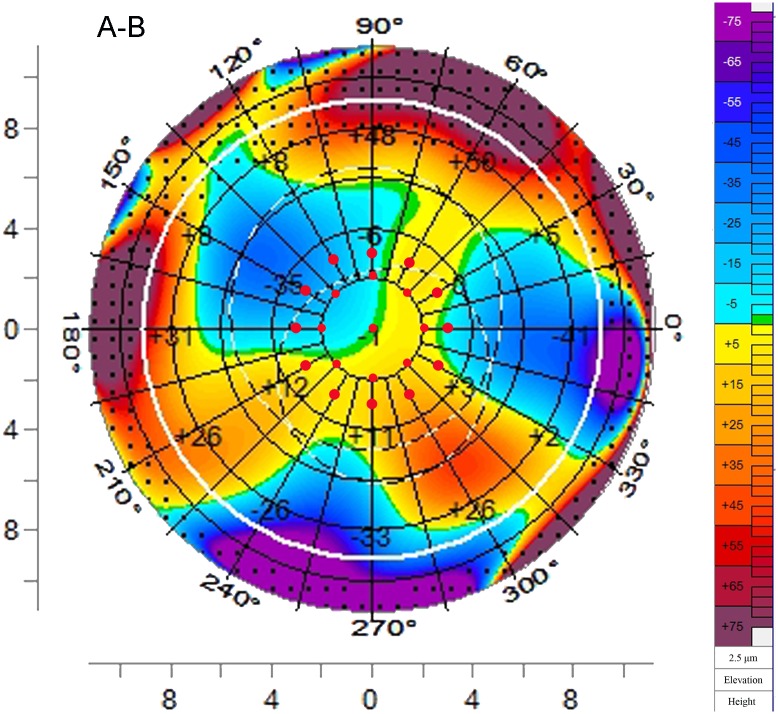
Changes of posterior surface elevation in the comparative map measured by Pentacam. Twenty-one points (red points) in the map were selected, including the corneal apex, eight points in the 4mm diameter annulus (0°, 45°, 90°, 135°, 180°, 225°, 270°, 315°semimeridians) and twelve points in the 6 mm diameter annulus (0°, 30°,60°,90°, 120°,150°,180°, 210°, 240°, 270°, 300°, 330°semimeridians).

PCE and changes by Pentacam were calculated at the corneal apex, the 4mm diameter annulus (0°, 45°, 90°, 135°, 180°,225°, 270°and 315°semimeridians), and the 6mm diameter annulus (0°, 30°,60°,90°, 120°,150°,180°, 210°, 240°,270°,300° and 330°semimeridians). We set 0° at a point on the right and moved counter clockwise. Changes in the posterior surface were determined by subtracting the postoperative elevation data from the preoperative elevation data using the comparative maps (Fig 2). An ectatic change resulted in a negative value.

### Strip-extensometry test

The two eyeballs of each rabbit were carefully extracted and washed with a balanced salt solution (BSS) to clean the hair and blood from the corneal surface. The eyeballs were kept at a temperature of 4° in a moist chamber for 12 hours before extensometry test. A dumbbell corneal strip with a width of 2mm was dissected from the centre region in the nasal temporal direction. BBS was instilled on the strip surface to maintain hydration. The cornea strip was clamped vertically with a distance of 3.5 mm between the jaws of a computer-controlled biomaterial tester (CMT6103, SANS Testing Machine Co., Ltd, Shenzhen, China). Strain was increased at a velocity of 12 mm/min until the tissue ruptured. The Young’s modulus was used to describe the material’s intrinsic biomechanical property. A high Young’s modulus indicates a stronger ability to defend against outside forces meaning a stiffer material. Young’s modulus was calculated as the chord between two points on the linear section of the stress-strain curve. [14]

### Statistical analysis

The Shapiro-Wilks test was used to verify the distribution of normality. Student’s t-test was used to compare the difference between the two surgery groups. Paired t-test was used to compare the changes between the preoperative and postoperative data. It was previously reported that corneal biomechanics were influenced by IOP [13,15–17], thus we compared the difference between two groups again after adjusting IOP using multiple linear regression. Single linear regressions were used to evaluate the correlation between CT or RBT and PCE changes or Young’s modulus to exclude the possibility of any influence of CT or RBT on PCE changes as well as Young’s modulus. All statistical tests were performed using SPSS version 20.0 (SPSS, Inc., Chicago, IL). A *P*-value<0.05 was regarded as significant.

## Results

Table 1 summarises the pre-operative manifest refraction spherical equivalent (MRSE), CCT, and RBT. There were no significant differences in manifest refraction and CCT between groups at baseline. The estimated RBT after surgery was thicker in the 100-cap group compared to 160-cap group as expect (*p* = 0.001). Little edema of the corneal incision was observed at the first day after surgery and disappeared within one week. Although the incision did not close spontaneously as in a human eye, no sign of infection was observed during the follow up.

**Table 1:** Preoperative refraction, central corneal thickness, and estimated thickness of residual stroma.

Data (Mean±SD)	100-cap group(n = 6)*	160-cap group(n = 6)*	P-value
Number of eyes	6	6	
Sphere, diopter	2.90±1.02	2.40±0.89	0.435
Cylinder, diopter	1.10±0.80	1.55±0.51	0.321
CCT, μm	366±25.85	367±22.95	0.972
Residual stromal thickness	188±25.41	128±22.42	**0.001**

* Cap thickness of SMILE;

n, number of eyes; CCT, central cornea thickness.

In the 100-cap group, DA and A2T significantly decreased one week after surgery (DA: *p* = 0.023; A2T: *p* = 0.042) compared to baseline values. The A1T was significantly decreased 3 months postoperatively in 160-cap group (*p =* 0.040). In the comparison of the two groups, A2T was longer in the 160-cap group (22.66±0.16ms) 4 month postoperatively than in the 100-cap group (21.75±0.29, *p* = 0.004). Neither DA nor A1T were significantly different between the two groups at each time point (Table 2). Multiple linear regression analysis found all of the differences were without significance after adjusting the IOP.

**Table 2:** Changes of corneal biomechanical metrics by Corvis ST for each depth SMILE.

Time point	100-cap group		160-cap group		P-value†	Adjusted
	Mean±SD	P-value*	Mean±SD	P-value*		P-vale‡
**DA, mm**						
Pre-operation	1.37±0.11	-	1.15±0.06	-	0.63	0.064
1 day	1.29±0.12	0.218	1.24±0.15	0.505	0.544	0.802
1 week	1.07±0.20	**0.023**	1.19±0.18	0.759	0.31	0.397
1 month	1.19±0.30	0.244	1.28±0.06	0.111	0.784	0.868
2 months	1.16±0.04	0.166	1.27±0.29	0.327	0.439	0.211
3 months	1.26±0.03	0.422	1.20±0.21	0.352	0.674	0.659
4 months	1.23±0.12	0.483	1.37±0.10	0.316	0.618	0.983
**A1T, ms**						
Pre-operation	6.31±0.16	-	7.00±0.14	-	0.117	**0.037**
1 day	6.80±0.51	0.088	6.61±0.13	0.649	0.465	0.598
1 week	6.95±0.62	0.073	6.61±0.54	0.511	0.418	0.946
1 month	7.09±0.72	0.136	6.64±0.21	0.317	0.629	0.139
2 months	6.55±0.19	0.722	6.66±0.46	0.373	0.444	0.83
3 months	6.49±0.11	0.511	6.75±0.33	**0.04**	0.468	0.611
4 months	6.77±0.21	0.176	6.49±1.00	0.227	0.212	0.573
**A2T, ms**						
Pre-operation	22.03±0.65	-	22.00±0.54	-	0.673	0.575
1 day	6.80±0.51	0.976	21.70±0.39	0.507	0.288	0.314
1 week	21.41±1.27	**0.042**	21.72±1.26	0.396	0.946	0.413
1 month	21.17±1.30	0.098	21.74±0.55	0.621	0.919	0.066
2 months	22.27±0.33	0.804	21.69±0.47	0.568	0.461	0.748
3 months	21.96±0.61	0.927	21.25±1.58	0.754	0.461	0.523
4 months	21.75±0.29	0.314	22.66±0.16	0.199	**0.004**	0.167

*P-value between pre-operation and post-operation.

^†^P-value between cap thickness of 100 μm and cap thickness of 160 μm.

^‡^P-valebetween cap thickness of 100 μm and cap thickness of 160 μm after adjusting intraocular pressure.

SMILE, femtosecond small incision lenticule extraction; Corvis ST, Corneal Visualization Scheimpflug Technology; SD, standard deviation.

Table 3 shows the changes of PCE in various optical zones at each follow-up time for each depth SMILE. The positive difference values at each time point for the two groups demonstrate that the postoperative posterior surface did not shift forward, in the 160-cap group, the apex even significantly shifted backward 3 months after surgery (*p* = 0.037). The displacement in the 160-cap group was not significantly different from that in the 100-cap group. Single linear regression analysis showed that neither CC nor RBT was related to the PCE changes in both groups explaining that PCE changes could not influenced by CC or RBT (Table 4).

**Table 3:** Changes of posterior surface elevation in various optical zones at each follow-up time for each depth SMILE.

Time point	100-cap group			160-cap group			Adjusted P-value†
	Mean±SD	Changes	P-value*	Mean±SD	Changes	P-value*	
**Corneal apex**							
Pre-operation	2.17±2.56	-	-	4.33±2.66	-	-	-
1 week	2.67±3.59	-0.50±2.88	0.688	7.80±6.53	-2.33±7.34	0.483	0.466
1 month	3.00±2.12	-1.50±2.81	0.255	3.00±5.92	0.50±6.72	0.606	0.514
2 months	1.00±3.16	0.17±2.71	0.895	3.40±2.70	0.17±3.25	0.491	0.868
3 months	1.00±1.41	0.17±1.33	0.789	-1.00±4.69	3.83±4.88	**0.037**	0.319
4 months	6.67±9.82	-3.00±7.35	0.423	-0.33±1.53	2.67±3.88	0.081	0.126
**4 mm annulus**							
Pre-operation	2.29±9.31	-	-	2.94±7.90	-	-	-
1 week	2.10±7.72	0.19±10.04	0.898	3.00±10.92	-0.17±10.47	0.913	0.763
1 month	2.33±6.95	-0.69±11.15	0.672	2.04±6.23	0.00±8.98	1	0.838
2 months	1.42±6.81	-0.21±12.03	0.906	1.85±5.87	0.19±8.17	0.875	0.857
3 months	1.06±4.38	0.15±9.82	0.919	0.21±7.80	1.83±10.92	0.253	0.265
4 months	2.67±5.98	-2.06±9.64	0.147	0.28±3.22	1.10±7.21	0.333	0.224
**6 mm annulus**							
Pre-operation	0.99±12.47	-	-	0.64±10.12	-	-	-
1 week	0.85±11.03	0.14±14.07	0.933	-0.64±16.59	1.33±15.98	0.482	0.992
1 month	1.03±8.92	-0.21±14.67	0.905	0.58±9.60	0.13±12.65	0.934	0.668
2 months	1.15±8.52	-0.65±15.29	0.72	0.33±7.90	0.38±11.10	0.776	0.716
3 months	1.03±5.64	-0.53±12.50	0.723	0.68±9.72	0.03±13.64	0.986	0.841
4 months	1.47±6.12	-1.42±11.35	0.297	0.35±4.51	0.14±9.28	0.901	0.302

SMILE, femtosecond small incision lenticule extraction; SD, standard deviation; 4mm, 4mm diameter annulus; 6mm, 6mm diameter annulus.

*P-value between pre-operation and post-operation.

^†^P-value of shift of posterior surface in each time point between two groups.

**Table 4:** Linear regression analyses of PCE changes and corneal biometric parameters.

PCE changes	Corneal cap thickness		Residual stromal thickness	
	β	P-value	β	P-value
**Corneal Apex**				
1 month	-0.042	0.554	0.035	0.538
4 months	-0.189	0.153	0.216	0.107
**4 mm annulus**				
1 month	-0.014	0.72	0.012	0.692
4 months	-0.121	0.315	0.138	0.193
**3 mm annulus**				
1 month	-0.022	0.182	0.008	0.519
4 months	-0.054	0.349	0.066	0.192

PCE, posterior corneal elevation

Changes of the ECD were not significantly different between the two groups at each observation point or during follow-up (Table 5). Young’s modulus was higher in 160-cap group than100-cap group, but with no statistical significance (160-cap group: 0.074±0.03 MPA, 100-cap group: 0.069±0.02 MPA; *p* = 0.077). Single regression analyses did not detect associations between Young’s modulus and RBT (β = 0.000, P = 0.412) or CT (β = 0.005, P = 0.810), indicating RBT and CT would not significantly affect the results of Young’s modulus.

**Table 5:** Changes of density of cornea endothelial cells for each depth SMILE.

Time point	100-cap group		160-cap group		P-value†
	Mean±SD	P-value*	Mean±SD	P-value*	
Pre-operation	994.6±8.8		995.8±3.6		0.77
1 week	986.3±6.4	0.06	989.4±17.4	0.505	0.668
1 month	992.7±16.1	0.642	994.5±9.2	0.719	0.849
2 months	993.3±2. 8	0.503	987.4±6.4	0.109	0.142
3 months	984.4±15.8	0.395	984.6±11.8	0.217	0.981
4 months	1001.0±4.4	0.677	983.3±6.0	0.137	0.181

*P-value for differences between pre-operation and post-operation.

^†^P-value for differences between two groups.

SMILE, femtosecond small incision lenticule extraction; SD, standard deviation.

## Discussion

SMILE is a newly developed refractive surgery that has advantages in preserving the integrity and biomechanical stability of the cornea. In this study, we found that changes of CST outputs, Young’s modulus, PCE, and ECD did not differ between SMILE performed at the depth of 100μm and 160 μm indicating that the possibility of a very large difference when using different CT during SMILE could be excluded.

McPhee et al.[18] demonstrated that tensile load acting (stress) on each layer through the thickness of rabbit cornea was similar and actual strain undergone was approximately equal in each layer. Thus, difference of Young’s modulus of a rabbit’s eye from the anterior to posterior part might be subtle. Our findings were consistent with recent retrospective case series in human. Güell et al. [19]compared the visual and refractive outcomes of SMILE with four different cap thicknesses (130μm, 140μm, 150μm, 160μm) and showed no difference between groups in corrected and uncorrected distant visual acuity, MRSE, Objective Scattering Index. In another retrospective study, El-Massry et al. [6]compared 100-cap and 160-cap SMILE and reported no significant difference in MRSE, uncorrected visual acuity, or total high-order aberration between groups.

Although we observed no statistically significant differences, it should be interpreted in the context of the magnitude of the hypothetical difference and the statistical power of the study given the number of eyes and sources of error (measurement method and variation between individual rabbit corneas). Based on the 0.08%/μm according to the model by Reinstein et al[5], this would predict a 6% difference between a 100 μm cap and a 160 μm cap. Interestingly, there was a 6.8% difference in Young’s modulus (0.069 and 0.074 MPA) in the present study, which was close to being statistically significant (p = 0.077). A power calculation for this difference showed that the 6 eye population would only enable 6% power and the study would require 410 eyes to be able to detect this difference with 80% power. In other words, the difference that is expected between a 100 and 160 μm cap is quite small (only 6%), so a 6 eye study with all of the potential errors associated with the measurements will not be able to detect it. A 6 eye study with 80% power would only be able to detect a 34% difference in Young’s modulus. In our study, the tensile tests were performed 4 months post-operation, the biomechanics might have partly recovered at this stage, which could also lead to a difficulty in detecting a significant difference between groups. In summary, this study excludes the possibility that there was a large difference when using different CT during SMILE surgeries.

Difficulty and complexity of the re-treatment of residual refractive errors represent the essential drawback of SMILE technique, in contrast to successful re-treatment procedures in PRK, LASIk and Epi-LASIK. Theoretically, the 160-cap SMILE will leave more space within the primary cap for secondary SMILE to correct residual refractive errors. Güell et al. [19] reported 2 cases underwent 160-cap SMILE firstly and 130-cap SMILE later with excellent visual and refractive outcomes, but more clinical trials with large sample size are still needed to confirm such conclusion.

The difference is noticed in 4 month for A2T at the raw analysis. However, after adjusting IOP, the statistical significance was abolished in the A2T at 4 months (P = 0.167). Our previous and other teams’ studies in human demonstrated corneal biomechanics were correlated with various demographic and biometrical factors. Several studies revealed association between A2T and IOP, which should be taken into consideration when interpretation of corneal biomechanics by Corvis ST. In addition, other parameters (11/12) provided by Corvis ST were not statistically significant in the data analysis. These indicated that corneal biomechanical differences were not significant on the whole. However, further study with larger sample size and longer follow-up are urgently needed to confirm or refute our assumptions.

A forward change in PCE is an early sign of ectasis and keratoconus. [20–22] We found neither significantly forward shift of posterior surface in both groups after SMILE nor significantly larger displacement in 160-cap group, suggesting SMILE with a thicker cap did not increase the risk for ectasis. Besides, our study showed that ECD was not influenced by deeper lenticule extraction. This was consistent with a previous report showing no change of ECD at 1 day or 1 year after SMILE. [23]

This study has limitations. First, the small sample size is a big weakness. Despite this, power analysis showed that a minimum 410 eyes will be needed to detect a difference, indicating no large difference existed. This implies that statistical power should not be a serious concern in our conclusion. However, a larger study is warranted to validate our pilot findings, and such a study with longer follow-up is being undertaken to update our analysis. Second, tensile experiments were easily influenced by several factors, such as the hydration level, curve shape and thickness of the corneal specimen.[24,25] Third, the conclusions might not be directly extrapolated to humans because structure of animal eyes differed greatly from that of human eyes. The Bowman’s membrane was absent in rabbit’s cornea, which may reduce Young’s modulus.[26,27] Future studies in human Eye Bank donor eyes are needed, since, for example, the average endothelial cell density in humans are 2000–2500, whereas in rabbits as in this paper, average ECD is around 950–1000. Finally, whether the parameters reported by Corvis device were adequate for biomechanical comparison needed to be confirmed. However, there is a lot of work on waveform analyses of deformation amplitude on going.

## Conclusion

In summary, the present study showed similar changes of corneal biomechanical metrics, PCE, and ECD between SMILE made at 100 and 160μm.This study could only exclude the possibility there was a very large difference when using different CT during SMILE surgeries. On the other hand, SMILE at deeper depth might provide a chance of secondary SMILE within the cap to correct residual refraction. Future clinical studies with larger sample sizes and longer duration are required to confirm our results.
